# Host Gene Expression Signatures Discriminate between Ferrets Infected with Genetically Similar H1N1 Strains

**DOI:** 10.1371/journal.pone.0040743

**Published:** 2012-07-13

**Authors:** Karl Ljungberg, Alexis McBrayer, Jeremy V. Camp, Yong-Kyu Chu, Ronald Tapp, Diana L. Noah, Sheila Grimes, Mary L. Proctor, Peter Liljeström, Colleen B. Jonsson, Carl E. Bruder

**Affiliations:** 1 Department of Microbiology, Tumor and Cell Biology, Karolinska Institutet, Stockholm, Sweden; 2 Southern Research Institute, Birmingham, Alabama, United States of America; 3 Department of Microbiology and Immunology, University of Louisville, Louisville, Kentucky, United States of America; 4 Research Resources Facilities, University of Louisville, Louisville, Kentucky, United States of America; University of Ottawa, Canada

## Abstract

Different respiratory viruses induce virus-specific gene expression in the host. Recent evidence, including those presented here, suggests that genetically related isolates of influenza virus induce strain-specific host gene regulation in several animal models. Here, we identified systemic strain-specific gene expression signatures in ferrets infected with pandemic influenza A/California/07/2009, A/Mexico/4482/2009 or seasonal influenza A/Brisbane/59/2007. Using uncorrelated shrunken centroid classification, we were able to accurately identify the infecting influenza strain with a combined gene expression profile of 10 selected genes, independent of the severity of disease. Another gene signature, consisting of 7 genes, could classify samples based on lung pathology. Furthermore, we identified a gene expression profile consisting of 31 probes that could classify samples based on both strain and severity of disease. Thus, we show that expression-based analysis of non-infected tissue enables distinction between genetically related influenza viruses as well as lung pathology. These results open for development of alternative tools for influenza diagnostics.

## Introduction

The disease caused by the pandemic H1N1 influenza strains (H1N1pdm) that emerged in 2009 differed from previously circulating seasonal strains. Although most patients displayed mild symptoms, the proportion of hospitalized and/or deceased healthy young adults was significantly higher than that seen in preceding influenza seasons [1], [2]. Recent evidence suggests that infection with genetically related H1N1pdm isolates results in widely different pathology [3], [4]. Current diagnostic approaches used in most hospitals do not routinely discern between influenza strains with minor genetic differences. Therefore, strains that confer a higher virulence in patients can initially be overlooked until a cluster of patients is noted with similar severity. To establish the exact infectious strain, current procedures require sampling of virus during shedding and then either sequencing or analysis using a set of strain-specific PCR primers, targeting the minute differences between strains. Diagnostic procedures based on gene expression profiling of non-infected blood cells have previously been used to discriminate between infections with different respiratory viruses as well as between symptomatic and asymptomatic subjects infected with influenza (H3N2) [5], [6]. Therefore, we hypothesized that strain-specific host gene response after infection with different, albeit genetically similar, influenza viruses would be sufficient to correctly identify the infectious strain using sophisticated statistical classification algorithms.

To test this hypothesis, we examined the systemic response to infection of two pandemic strains (A/California/07/2009 and A/Mexico/4482/2009) and compared these responses to those triggered by a seasonal H1N1 strain (A/Brisbane/59/2007) in blood samples from infected ferrets using a ferret-specific microarray [7]. The domestic ferret is susceptible to most human influenza isolates and develops clinical symptoms resembling those seen in humans [8]. It should however be noted that the pathology observed in human subjects is not always reflected in ferrets infected with the same strain.

The analysis revealed that H1N1 infection in ferrets influenced transcription of common functional gene clusters involved in processes such as lysosomal protein degradation, virus response, and apoptosis control in blood cells. However, the bulk of the identified expression changes showed strain-specific patterns. We utilized these strain-specific alterations to delineate the smallest number of genes needed to identify the infectious strain and/or severity of disease.

## Results

### Clinical Evaluation, Virology and Histopathology

**Figure 1:**
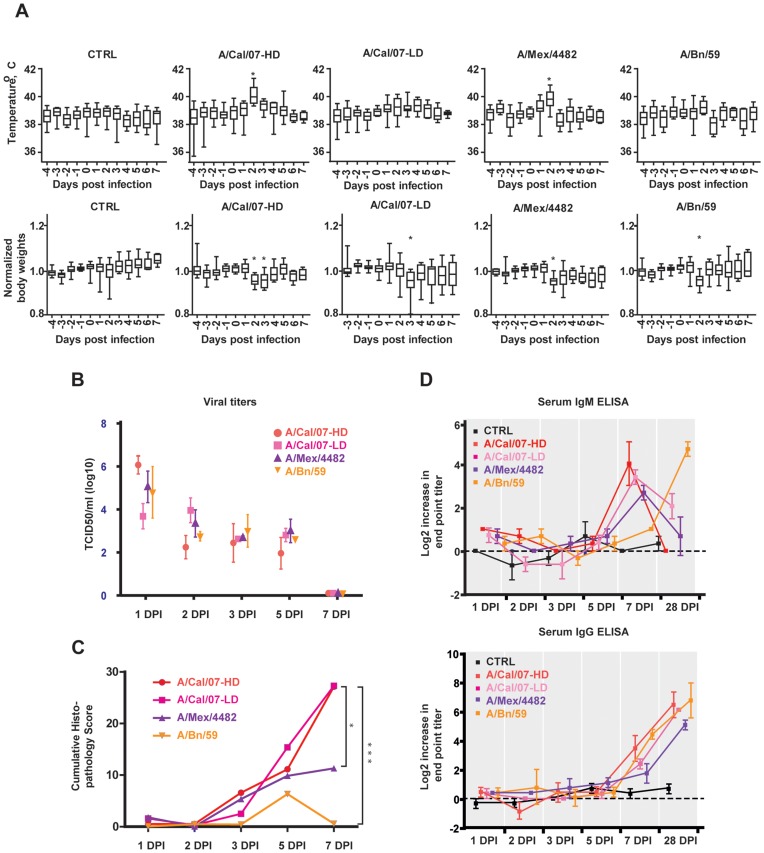
Clinical evaluation, virology and immunology. Box-and-whisker diagram of the body temperature and body weight (A) for the controls and the infected animals. The stars indicate significant increases of body temperature and significant loss of body weight (P<0.001, ANOVA followed by Bonferroni’s multiple comparison test). The temperature and body weight recorded prior to infection was used as normal level. Panel B shows the viral titers in the nasal turbinates. Significant difference in titers between strains were obtained at 1 DPI (A/Cal/07-HD vs. A/BN/59; A/Cal/07-LD vs. A/Mex/4482) and at 2 DPI (A/Cal/07-LD vs. A/BN/59) (ANOVA, p<0.05, Bonferroni correction). Panel C shows the log2 increase in end point titers of IgM and IgG between serum obtained prior to infection and at the time of euthanasia for each animal.

The clinical signs began 2 DPI, and resolved at 5 DPI for A/Cal/07 and A/Bn/59 and at 6 DPI for A/Mex/4482. Sneezing, nasal and ocular discharges were observed in all groups except the control. The animals infected with the A/Cal/07-HD or A/Mex/4482 had significantly increased body temperatures at 2 DPI (*P*<0.0001, ANOVA followed by Bonferroni’s multiple comparison test) as shown in Figure 1A. A/Bn/59 and A/Cal/07-LD infected ferrets did not show significantly increased body temperatures. A significant decrease in body weight as compared to uninfected controls was detected at 2 and 3 DPI in A/Cal/07-HD infected and at 3 DPI in A/Cal/07-LD infected ferrets (P<0.001). Body weight loss was also seen in animals infected with A/Mex/4482 and A/Bn/59 at 2 DPI only (P<0.001). By 28 DPI, all animals had returned to the weight recorded at 0 DPI.

We analyzed nasal swabs, nasal turbinates and lungs for presence of virus and confirmed that all three strains of influenza established infection (Fig. 1B). The highest titers were detected in the nasal swabs on 2 DPI in all groups except for A/Cal/07-LD, which peaked at 3 DPI. The highest titers recorded were 10^3.8^ TCID_50_/ml in A/Cal/07-HD, 10^4.5^ TCID_50_/ml in A/Mex/4482 and 10^2.3^ TCID_50_/ml in A/Bn/59 and 10^3.4^ TCID_50_/ml for A/Cal/07-LD. Similar results were obtained for nasal turbinates. Virus could also be isolated from lungs from euthanized ferrets. Virus production peaked at 3 DPI (A/Cal/07-HD, 10^2.3^ TCID_50_/ml; A/Mex/4482, 10^2.2^ TCID_50_/ml and A/Bn/59, 10^2.5^ TCID_50_/ml) for all groups but A/Cal/07-LD. The peak for this group was seen at 5 DPI (10^2.3^ TCID_50_/ml). Influenza virus was not detected in any sample by 7 DPI. In addition, no virus was detected in samples obtained from brain, jejunum, colon or liver collected at 3 DPI.

Histopathological evaluation identified lesions in the airways and lungs (Table S1 and Fig. S1). Microscopic lesions observed in lung tissue were compatible with viral infection and inflammation. Lesions were found in animals infected with any of the three strains. The incidence and severity of microscopic lesions in the H1N1pdm infected animals generally increased over time (Fig. 1C). Lung pathology based on cumulative histopathology score was most severe in A/Cal/07-HD infected animals, followed by the A/Cal/07-LD infected ferrets. The cumulative histopathology score in these two groups were significantly higher than the A/Bn/59 infected animals at 7 DPI (ANOVA, p<0.05 followed by Bonferroni’s multiple comparison test). The animals infected with A/Mex/4482 showed a higher, but not significant, cumulative histopathology score than the A/Bn/59 infected ferrets. By 28 DPI the pulmonary lesions were resolved in all groups.

Influenza-specific IgM followed by Influenza-specific IgG production was detected in all animals (Fig. 1D). An early peak in IgM followed by IgG production is consistent with the kinetics of immunoglobulin production in response to an infection.

### Statistical and Functional Clustering Analysis of Gene Expression Data

Prior to statistical analysis, correlation coefficients were calculated between each sample within each group. The overall correlation between each sample was high (94% or higher, Fig. S2A). In order to elucidate a possible batch effect, we used unsupervised nonnegative matrix factorization (NMF) [9]. Since we used three different strains and a control group, we performed an NMF clustering of the samples into four ranks. Three of the four clusters contained samples from more than one strain, whereas one cluster was composed on samples from the A/Cal/07 infected group only. No obvious clustering was seen based on methodological parameters, like hybridization date and slide number (Fig. S2B). Variance analysis of the microarray data from the blood samples identified 4098 of 30742 probes that were significantly altered when grouping the animals according to euthanasia day, infectious strain and dose (ANOVA, p-value <0.01, Bonferroni FDR correction). As a number of genes on the array were represented by multiple probes, we used CAP3 [10] to identify probes with sequences that were overlapping by 90% or more. These probes showed nearly identical expression patterns (data not shown), and thus only one probe for each such gene was used for further analysis. After CAP3 filtering, 3232 probes remained.

**Figure 2:**
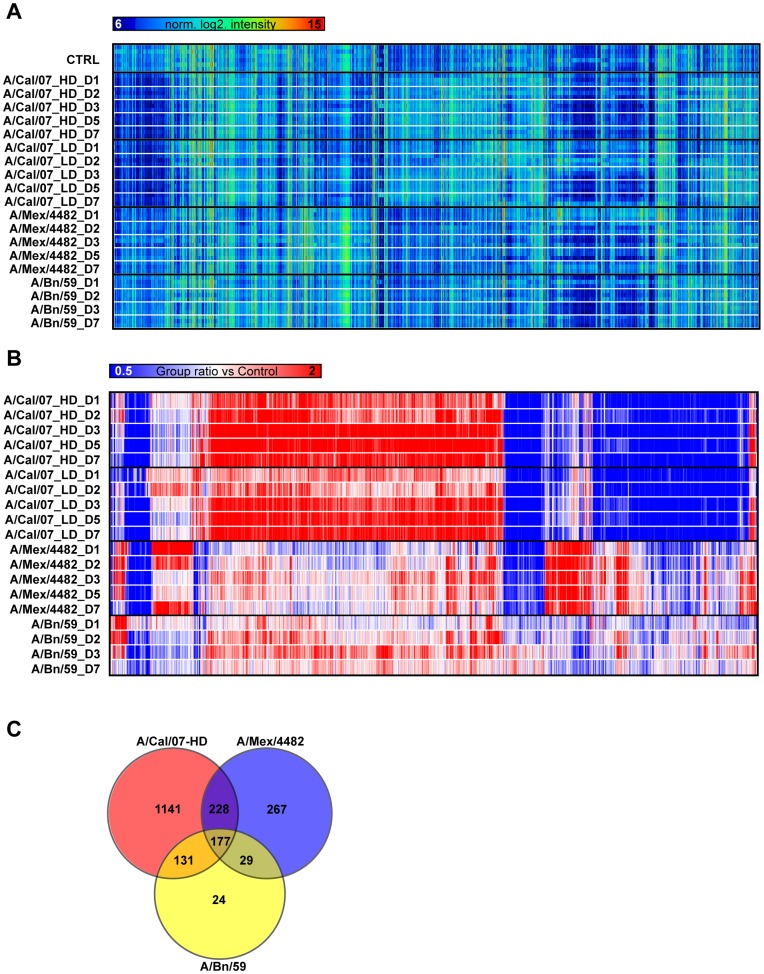
Global gene expression changes. Panel A shows a heat map of the 25% (7685 probes) most variable genes in the dataset for all samples. Each sample day represents three individual animals, except the control group (CTRL, n = 6). HD and LD indicate high dose and low dose, respectively. D1 through D7 designates the day of euthanasia. Panel B illustrates gene expression profiles of 1997 significantly changed probes with a fold change larger than +/−2 in at least one group when compared to the control group. The average fold change from the three animals within each group is shown. Red designate up-regulated genes, blue down regulated genes, where a more intense color illustrates a more pronounced fold change. The Venn diagram in panel C shows the number of probes up or down regulated after infection by any of the three strains (177 genes), by two of the three strains (228, 131 and 29 genes) and the number of probes aberrantly expressed in a strain specific pattern.

Of these, 1997 probes displayed a fold change ratio that was larger than +/− two-fold for at least one time point for any of the three influenza strains (Fig. 2). These probes represented 1200 annotated genes or transcripts. There were 1050 probes that could be mapped against human orthologs using the Entrez gene database, and these genes were clustered based on their biological function using DAVID. Protein localization (GO: 0008104) and phosphorous metabolic process (GO: 0006793) ranked highest with 98 and 93 genes respectively. As expected, many of the genes altered compared to the control animals were part of the regulation of cell death (GO: 0010941, 77 genes) and immune response (GO: 0006955, 72 genes).

Of the 177 probes that were commonly up- or down regulated by all strains, 72 had human orthologs and were further analyzed using DAVID to highlight involved biological processes. The highest ranked gene ontology categories were immune response (GO: 0006955) and apoptosis (GO: 0006915) (data not shown). The majority of the altered genes did however show a strain-specific expression pattern, and animals infected with A/Cal/07 displayed a larger fraction of altered probes compared to A/Mex/4482 and A/Bn/59 (Fig. 1C). Confirmatory analysis using CYBR green qRT-PCR was done on a limited number of genes (*PTPRC, DDX58, CD36* and *MT-APT6*), which showed levels similar to those identified by the arrays (Fig. S3 and data not shown).

### Identification of Gene Expression Profiles Capable of Classifying Influenza Strain

**Figure 3:**
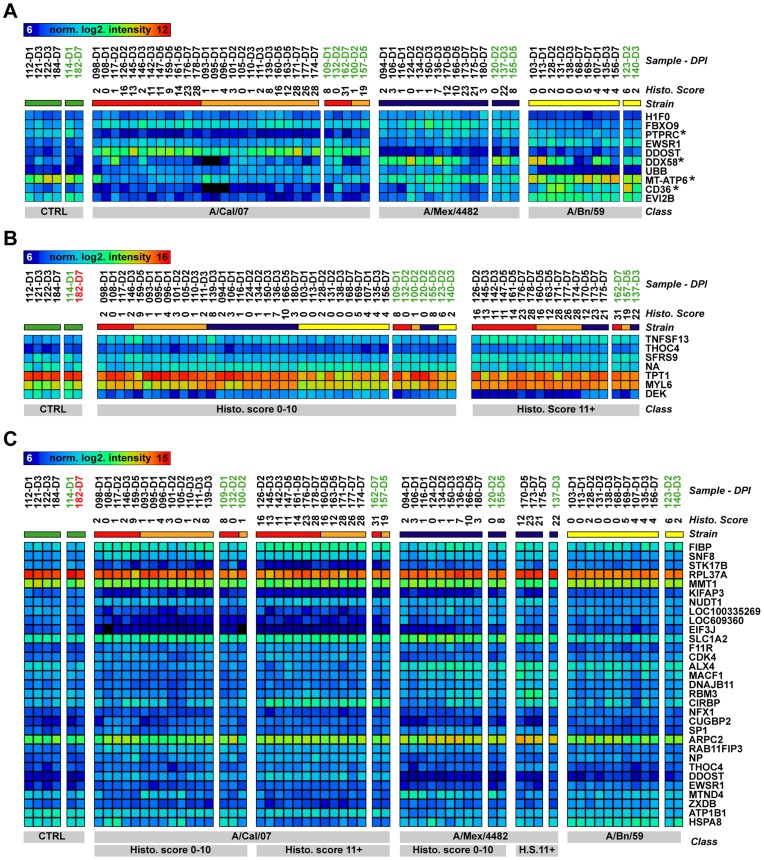
Classification analysis. Panel A shows the heat map of the 10 genes isolated by the USC algorithm to classify the samples with regards to infectious strain. The samples are denoted by their ID number and the euthanasia day, and are sorted according to strain (and dose for A/Cal/07 infected animals). The asterisks denote gene expression verified by qRT-PCR. Panel B shows the 7 genes that were used to classify the samples based on the cumulative histopathology score. The samples are denoted with ID number and euthanasia day, and are sorted according to cumulative histopathology score (given as numbers under the sample names). Panel C displays the 31 genes required to classify the samples based on infectious strain and histopathology score. The samples are denoted with ID number and euthanasia day, and are sorted according to strain and cumulative histopathology score (given as numbers under the heat map). All samples were correctly classified with regards to strain. The samples used to train the classification algorithm is denoted in black, correctly classified samples in the test set in green and incorrectly classified samples in red.

Hierarchical clustering of the 1997 probes separated the samples into three major clusters (A/Cal/07 infected animals in cluster 1, A/Mex/4482 infected animals in cluster 2 and A/Bn/59 and CTRL animals in cluster 3, data not shown). We thus hypothesized that we could identify a small set of genes capable of classifying the influenza strain used to infect the ferrets. We used uncorrelated shrunken centroid (USC) classification, as it provides a supervised learning approach by which the known classes or groups of samples are used to train the algorithm to isolate the smallest number of genes capable of categorizing an unknown sample [11]. To avoid over-fitting the algorithm, we divided the samples into a training set, containing 51 of the samples, and a test set with the remaining 12 samples (Fig. 3). The training set was used by the USC algorithm to identify the smallest number of “classifier genes”. These classifier genes were then used to identify the classes of the test set.

Using the genes that were significantly altered between the different strains (ANOVA, p-value <0.01, Bonferroni FDR correction) with the training set, the USC algorithm identified an expression profile consisting of 10 genes. Several of these genes have been implicated in innate immune response (F box protein 9 (*FBXO9*), protein tyrosine phosphatase, receptor type, C (*PTPRC* or *CD45*), ecotropic viral integration site 2B (*EVI2B* or *CD361*), CD36 molecule (thrombospondin receptor) (*CD36*) and DEAD (Asp-Glu-Ala-Asp) box polypeptide 58 (DDX58 or RIG-I) [12]–[16]. This classifier profile was then used to identify the infectious strain of the samples in the test set. The infectious strain was correctly classified in all samples this set of 10 genes (Fig. 3). Ten genes selected randomly using the sampling function in Microsoft Excel could not distinguish between the strains with high accuracy. Using these ten genes, 6 of 12 samples (50%) were incorrectly classified (data not shown).

We also tested if the expression profile of the 10 genes selected as strain classifiers were capable of categorizing samples generated in data generated elsewhere [17]. Here, gene expression profiles from A/Cal/07 and A/Bn/59 infected ferret lung samples generated through cross-species hybridization to a canine-specific microarray were obtained from the GEO omnibus database (Acc. no. GSE17079). We isolated 16 probe sets corresponding to the 10 genes used to classify the strains in our data set. We then created a training set with four of the six control samples and six of the nine A/Cal/07 and A/Bn/59 infected samples, respectively. The test set contained two control samples and three samples from each of the infected groups. Using the training set to first build the classifier, and then using this to identify the infectious strain in the test set, resulted in correct classification of 7 of out of 8 samples. One A/Cal/07 sample was classified as an A/Bn/59 infected animal (data not shown).

### Identification of Genetic Expression Profiles for Classification of Lung Pathology

To investigate if there were any gene expression profiles that correlated to lung pathology, we isolated the smallest number of genes that could classify the cumulative histopathology score. The samples in the training set were divided based on the cumulative histopathology score. The cutoffs were set to less or equal to ten, or eleven or higher. The USC algorithm then identified a gene profile capable of predicting the severity of lung pathology, independent of infectious strain. With a combined expression profile of seven genes, correct classification was obtained for all but one sample in the test set, (Fig. 3B). The genes used to classify the samples were: tumor protein, translationally-controlled 1 (TPT1); nucleoporin 210 kDa (*NUP210*); DEK oncogene (*DEK*); THO complex 4 (*THOC4*); splicing factor, arginine/serine-rich 9 (*SRSF9*); myosin, light chain 6 (*MYL6*) and tumor necrosis factor (ligand) superfamily, member 13 (*TNFSF13* or *LIGHT*). None of these genes overlapped with those used to classify the infectious strain.

We also isolated the smallest number of genes that could classify the samples with regards to strain and cumulative histopathology score. In this analysis, all samples were grouped based on the infectious strain as well as the cumulative histopathology score (CTRL, A/Cal/07_Hist0–10, A/Cal/07_Hist11+, A/Mex/4482_Hist0–10, A/Mex/4482_Hist11+ and A/Bn/59_Hist0–9). USC classification using the training set isolated 31 probes, which were able to correctly classify all but one sample in the corresponding test set. One control animal was erroneously classified as an A/Bn/59_Hist0–9 sample (Fig. 3C).

## Discussion

In April 2009, a new strain of influenza A H1N1 was identified in Mexico. The infection then spread rapidly around the world, and was declared a pandemic by the World Health Organization (WHO) in June 2009. The pandemic alert lasted until August 2010. Analysis of epidemiological data has indicated that several risk factors (such as obesity or high age etc.) for development of severe disease exist. Nevertheless, a remarkably large fraction of severely affected patients during the most recent influenza pandemic were among young and healthy individuals [1], [2], [18]. The explanation for this is currently not known, although recent evidence, including those presented here, suggests that genetically related influenza isolates can promote different host responses as well as clinical manifestations [3], [4], [19].

Commercially available, widely used rapid influenza diagnostic tests do not distinguish among influenza A virus subtypes and are less sensitive than viral culture or RT-PCR [20]. However, viral culture or RT-PCR has limitations, as they require isolation of replicating virus. The 2009 pandemic influenza strains follow standard influenza viral kinetics in humans. After an initial peak in viral load occurring 2 to 3 days after infection, virus titers decline and are undetectable after five days [21]. Several reports have indicated that the day from onset of symptoms to hospital admission varies from 0–22 days [22]–[24]. Thus, patients may come to the clinic after viral shedding has ceased, and current influenza diagnostics may thus fail to identify the exact infectious influenza strain. Given the fact that genetically related H1N1pdm isolates cause a wide range of clinical manifestations, identification of patients infected with more virulent strains or at risk of developing a more severe disease is of clinical relevance. Such information can facilitate decision-making regarding need for treatment vs. no need for treatment for individual patients, and has thus life-saving potential.

Recently, host gene expression profiling has been used to distinguish different respiratory viruses from patients infected with rhinovirus, respiratory syncytial virus or influenza A virus [5]. In addition, Huang et. al used gene expression profiling of peripheral blood cells to study the temporal dynamics of host response in human subjects infected with influenza A virus (A/Wisconsin/67/2005), and identified gene expression profiles that could discriminate between symptomatic and asymptomatic subjects [6]. To investigate if strain-specific gene expression signatures derived from infection with genetically similar influenza viruses could be used to identify the viral etiology of infection, we examined the systemic host response in peripheral blood samples from ferrets infected with three A/H1N1 influenza viruses. The majority of the transcriptional changes displayed a strain-specific induction or reduction, despite up to 99.88% sequence similarity between the strains. Previous studies have shown that influenza infection causes an increase of immune cells in the blood, and that cytokines such as IL-6, IL-8 and MCP-1 protein levels differ significantly in macaques infected with different H1N1pdm strains [3]. Different infection-induced protein levels in blood are likely connected to differential gene expression. It is thus not surprising that we detected different transcription profiles in the blood from ferrets infected with different influenza strains.

Several different patterns of this strain-specific host response were observed, including genes that were up- or down regulated as a result of infection by one of the strains examined, but not the other two. Alternatively, there were genes that were up- or down regulated in all infected animals, albeit the induction or reduction was several-fold different between the different strains. The homogenous transcription profiles among the animals within each group, and the large heterogeneity between the groups allowed for identification of strain-specific gene expression signatures.

Using a supervised classification algorithm, we could identify 10 genes whose collective expression profile was capable of correctly classifying all samples in a test set, where 12 of the samples in this study had been deliberately set as “unknown”, and had not been used to train the classification algorithm. Several of the genes in this profile have previously been shown to be involved in innate immune response, such as *DDX58, CD45, CD36* and *EVI2B.* It is interesting to note that the different influenza strains used in this study triggered a highly strain specific host response, also involving genes transcribing key pattern recognition molecules for influenza like the DDX58/RIG-I. This gene signature was further tested using lung samples derived from influenza infected ferrets generated by other investigators [17]. All but one sample in the test group were correctly classified, even though the data had been generated using lung tissue on another microarray platform. Hence, the signatures identified here were not limited to our ferret data.

We further used the classification algorithm to identify gene expression changes that associated with lung pathology. We identified 7 genes whose combined gene expression profile could classify all but one sample according to their cumulative histopathology score. One of these genes, *TNFSF14* encodes for a tumor necrosis factor (TNF) ligand superfamily member which has previously been shown to be involved in regulation of dendritic cells and stimulation of T cell proliferation [25]. Constitutive expression of *TNFSF14* has been shown to result in T cell mediated inflammation and tissue destruction. Myosin, light chain 6, alkali, smooth muscle and non-muscle (MYL6), has been indicated as a protein involved in cell migration, a key process in regeneration of damaged tissue [26]. THO complex 4 (THOC4) and serine/arginine-rich splicing factor 9 (SRFS9) are both involved in mRNA processing. The influenza virus utilizes several key host components for mRNA splicing and export, and it is thus interesting to note that genes involved in these mechanisms were expressed at higher levels in animals with a more severe disease. It remains to be investigated if high expression values of these genes are also present in human patients with severe influenza syndromes.

It is likely that both host factors as well as the infecting strain will determine the severity of disease in an individual patient. Thus, a comprehensive diagnostic test should ultimately be able to diagnose both the infecting strain and predict the severity of the disease in a single step analysis. Strain information is essential to identify patients infected with viruses that are resistant to available antiviral drugs. Similarly, identification of patients with higher risk of developing severe disease would help health care professionals in providing medical attention where it matters the most. Therefore, we tested if we could classify our samples with regard to strain and severity of lung pathology. Using a profile containing of 31 genes, we could classify all samples but one sample in the test set with regard to strain and histopathology score.

It is plausible that the severity of the disease is dependent on both host factors and the virulence of the infecting virus. Our study was not designed to identify a gene profile capable of predicting the clinical outcome, as some animals were euthanized prior to onset of disease. In addition, all our samples were not analyzed at one single time point, and were thus not fully randomized. This may induce variability that is of non-biological origin, although the NMF analysis done here argues against this. Also, since ferrets are outbred, our sample groups were not necessarily large enough to capture possible inter-individual ability to respond to the infection. To identify genes with a higher predictive power, a repeated sampling approach in a larger set of animals should be used, preferentially with multiple strains and multiple doses. Nevertheless, the results obtained here further expand on the findings by Zaas et al. where it was established that expression-based diagnostics could differentiate between several respiratory viruses [5], and shows promise for further development of a diagnostic tool that could be used for early identification of patients infected with highly pathogenic strains and facilitate early treatment as well as isolation to prevent transmission of virulent virus. Whether the combined expression profile of the genes used to classify the ferret samples are capable of classifying samples from human influenza patients’ needs to be further elucidated. However, based on our results and the analysis of host response to different H1N1pdm viruses in rhesus macaques [3], it can be extrapolated that genetically similar influenza viruses result in strain-specific host responses in humans. For clinical development, this approach needs to be validated in human blood samples. Additional analysis studying the overlap of gene expression changes between species infected with the same influenza strains is needed. In conclusion, we believe the results presented here open new avenues for precise molecular diagnostic tools capable of predicting the clinical outcome of influenza-infected patients.

## Materials and Methods

### Virus Preparation and Titer Determination

Viral stocks of the three H1N1 influenza strains A/California/07/2009, A/Mexico/4482/2009 and A/Brisbane/59/2007 (A/Cal/07, A/Mex/4482 and A/Bn/59) were obtained from the Centers for Disease Control and Prevention. The viruses were propagated for two to four days at 34°C in ten day old embryonated hen’s eggs according to standard operating procedures previously described [27], [28]. TCID_50_ analysis in Madin-Darby Canine Kidney (MDCK) cells was performed to assess the viral load in swabs and tissues as has previously been described [28].

### Animal Care, Clinical and Microscopical Evaluation and Sample Collection

All procedures were conducted in accordance with the Animal Welfare Act and the CDC-NIH Biosafety in Microbiological and Biomedical Laboratories and were approved by the Institutional Biosafety Committee and Institutional Animal Care and Use Committee (ACUP protocol #08-05-031B). The animal experiments were performed in the AAALAC-accredited ABSL-2 and ABSL-3 facilities at Southern Research Institute.

Castrated three to six months old ferrets (500–1800 g) were used (Triple F Farms). All animals were seronegative for representative currently circulating human influenza A strains as determined by hemagglutination inhibition assay. The ferrets were divided into groups defined by the strain used for infection and the day of euthanasia. The animals had free access to water and food and a maximum of two animals were housed per cage. All groups were housed in separate rooms. On Day 0, each ferret was anesthetized using a ketamine/xylazine/atropine mixture, formulated to provide doses of 25 mg/kg ketamine, 1.7 mg/kg xylazine, and 0.05 mg/kg of atropine to each animal, and challenged intranasally with one ml of 10^6^ TCID_50_/ml [A/Cal/07 high dose (HD), A/Mex/4482, and A/Bn/59] or one ml of 10^4^ TCID_50_/ml [A/Cal/07 low dose (LD)] virus (0.5 ml per naris) diluted in PBS. The mock-infected animals were challenged with PBS only.

Body weight, temperature and clinical signs of infection such as nasal and ocular discharge, sneezing, presence of loose stool, and inactivity were recorded daily. A post-mortem examination was performed on the day of euthanasia. Lungs, nasal turbinates, brain, jejunum, colon, and liver were collected for analysis of virus titers. In addition, lung samples were placed in 10% neutral buffered formalin for histopathological examination. Hematoxylin and eosin stained lung tissue was evaluated microscopically from each animal. Microscopic Pulmonary lesions were graded for severity using a numerical scoring system in which 1 = minimal, 2 = mild, 3 = moderate, and 4 = marked. The scores for each animal were then summarized to generate the cumulative histopathology score. Two ml of whole blood from each animal was collected in PaxGene RNA tubes for the microarray analysis.

### IgM and IgG ELISA

Serum was collected two weeks before infection and at 1, 2, 3, 5, 7, and 28 days post infection (DPI) and evaluated using virus-specific ferret IgM and IgG ELISA. Plates were coated with 1∶200 dilution of stock virus in PBS overnight at 4°C, and then blocked with 2% donor goat serum (Sigma Aldrich) in PBS/0.05% v/v Tween-20 for 30 min. Ferret serum was then added and 2-fold serially diluted and incubated at 4°C overnight. HRP-conjugated anti-ferret IgM or IgG was added and incubated at 37°C (1 h). TMB substrate was added and the reaction was stopped using 1 M H_3_PO_4_, and read at 450 nm.

### RNA Preparation, Labeling and Microarray Analysis

Blood from three animals per group was collected upon euthanasia at 1, 2, 3, 5 and 7 DPI. For A/Bn/59, the samples collected at 5 DPI did not meet the quality threshold, and were discarded from the analysis. The blood collected in PaxGene tubes was processed using the PaxGene RNA kit, according to the protocol supplied (Qiagen). Quantity and quality of the total RNA was analyzed using a NanoDrop 2000C Spectrophotometer (Thermo Scientific) and an Experion automated electrophoresis system (Bio-Rad) prior to labeling. To optimize detection of critical host responsive genes and avoid the possibility of identifying false positive and negative expression signals using cross-species microarray hybridization [29], [30], we used a ferret-specific microarray that we recently developed [7]. 200 ng total RNA was labeled using the one-color labeling kit from Agilent technologies according to the supplied protocol. The labeled cRNA was then hybridized on a previously described ferret specific microarray for 16 h and washed according to the supplier’s instructions. The slides were scanned at a GenePix 4000B scanner at five µm resolution. The raw image files were then processed by the Agilent feature extraction software and raw data files were obtained. The samples were analyzed in different cohorts. RNA from animals infected with A/Cal/07-HD and A/Cal/07-LD and the controls were hybridized first, followed by the A/BN/59 and lastly the A/Mex/4482. The raw data files from all samples were then normalized and filtered using the Agi4×44PreProcess R-plugin available at the bioconductor website. Agi4×44 performed inter-microarray normalization (quantile normalization) and filtered out spots below the low signal threshold (Fig. S2). The data was then log2 transformed and imported into Multi experiment viewer (MEV) v4.6 for statistical analysis [31]. Fold changes (FC) were calculated using Microsoft Excel and Access was used to combine results from the statistical tests. The raw data was deposited at the Gene Expression Omnibus data repository with access number GSE28967.

Functional analysis was done using the Database for Annotation, Visualization and Integrated Discovery (DAVID), [32]). DAVID performs batch annotation enrichment analysis to identify frequently co-occurring biological functions, protein-protein interactions, protein functional domains, disease associations, pathways etc. in a set of genes identified e.g. as up or down regulated in expression analysis. Concomitant gene clusters were ranked by statistical significance to highlight the most relevant biological functions. As no ferret genomic background was available, we used the human genome as background for the functional annotation investigations.

We used uncorrelated shrunken centroid classification (USC) to identify the smallest number of genes capable of categorizing an unknown blood sample with regard to which influenza strain had caused infection. This algorithm utilizes a supervised learning approach, in which the known classes or groups of samples are used to train the algorithm. USC then identifies a subset of genes which are then used to predict which group any unknown sample belongs to [11]. For the classifications, a training set containing 51 of the 63 samples was used to train the algorithm. The validity of the isolated genes was then tested using a test set, containing 12 samples intentionally left out during the training. In addition, we used microarray data from a previously published study [17], [33], [34] to confirm that the genes identified by the USC algorithm also could classify influenza infected ferrets analyzed elsewhere. These data sets were downloaded from the GEO omnibus repository (acc. no. GSE17079), normalized using the PLIER PM option in the Expression Console software from Affymetrix. Since this study utilized a different platform, we had to create a new training set containing eighteen of the samples and a test set contining the remaining eight samples from this study. The algorithm was trained using the probes sets that overlapped with the genes selected from our study, and was then used to classify the samples in the test set.

## Supporting Information

Figure S1 **Representative findings from the histopathological examination of lung tissue.** Examples of findings from the histopathological examination of the influenza infected ferrets. Panel A shows an example of chronic active perivascular inflammation in a A/Cal/07 infected animal (black arrows), Panel B indicate chronic active inflammation within the bronchiolar lumen (orange arrows) and bronchiolar hypertrophy and regeneration (black arrows). C illustrates bronchiolar hyperplasia (green arrows), inflammation of the bronchiolar wall (black arrows), perivascular interstitium (white arrow) and alveoli (orange arrow). In panel D, chronic active inflammation of alveoli and bronchiolar lumen is seen at the orange and the green arrow, respectively. The white arrows indicate bronchiolar necrosis. Panel E shows the geometric mean of cumulative histopathology score for each strain and euthanasia day.
(TIF)

Figure S2 **Normalization of microarray data and principal component analysis of samples.** Box-and-whisker diagram of the microarray intensities before (A) and after normalization (B). The average correlation coefficient for the samples within each group is shown above the diagram in panel B. Panel C shows the consensus matrix of an unsupervised Nonnegative matrix factorization (NMF) analysis, using the entire data set prior to any statistical comparisons. The color of the heat map indicates the cophenetic correlation used to quantify the robustness of the rank’s evaluation. A strong correlation is indicated as black and weak correlation as red. Strong correlation between the A/Cal/07 infected animals was seen, whereas the other animals did not form any obvious clusters. No obvious clustering could be attributed to be the experimentally introduced variability.
(TIF)

Figure S3 **qRT-PCR validation.** Scatter plots showing the ΔΔ−C_t_ values (blue diamonds) and the fold change ratio from the microarray (red circles) for the *PTPRC* and *MT-APT6* genes in control samples and A/Cal/07 infected samples.
(TIF)

Table S1 **Lung histopathology results.** Microscopic Pulmonary lesions were graded for severity using a numerical scoring system in which 1 = minimal, 2 = mild, 3 = moderate, and 4 = marked. The scores for each animal were then summarized to generate the cumulative histopathology score.
(XLSX)
