# Supplementary figures and images for: Host Gene Expression Signatures Discriminate between Ferrets Infected with Genetically Similar H1N1 Strains
Source: PLoS One. 2012 Jul 13;7(7):e40743. doi: 10.1371/journal.pone.0040743 (PMC3396591; doi:10.1371/journal.pone.0040743)

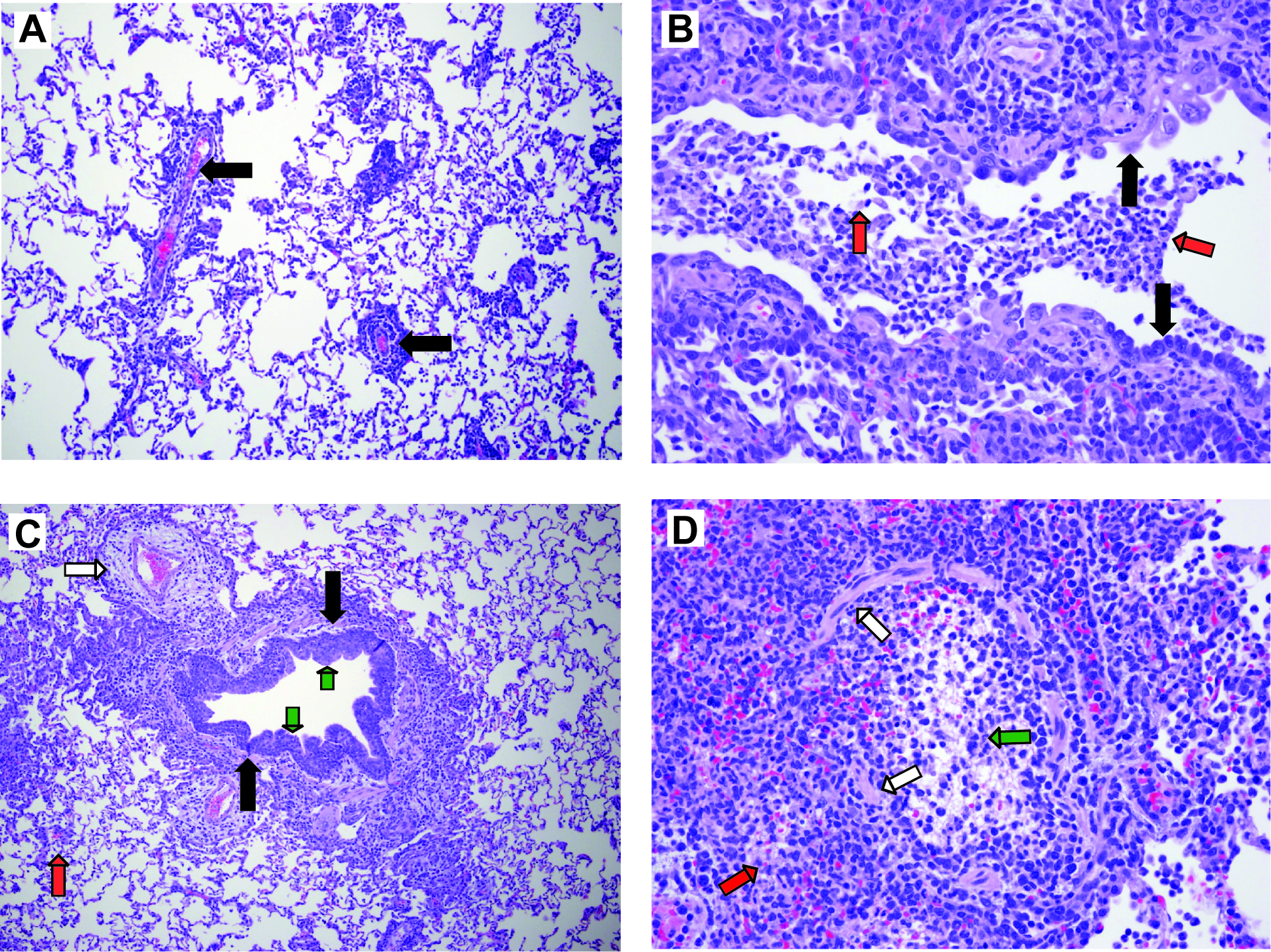

Supplement: Figure S1 — Representative findings from the histopathological examination of lung tissue. Examples of findings from the histopathological examination of the influenza infected ferrets. Panel A shows an example of chronic active perivascular inflammation in a A/Cal/07 infected animal (black arrows), Panel B indicate chronic active inflammation within the bronchiolar lumen (orange arrows) and bronchiolar hypertrophy and regeneration (black arrows). C illustrates bronchiolar hyperplasia (green arrows), inflammation of the bronchiolar wall (black arrows), perivascular interstitium (white arrow) and alveoli (orange arrow). In panel D, chronic active inflammation of alveoli and bronchiolar lumen is seen at the orange and the green arrow, respectively. The white arrows indicate bronchiolar necrosis. Panel E shows the geometric mean of cumulative histopathology score for each strain and euthanasia day. (TIF) [file pone.0040743.s001.tif]

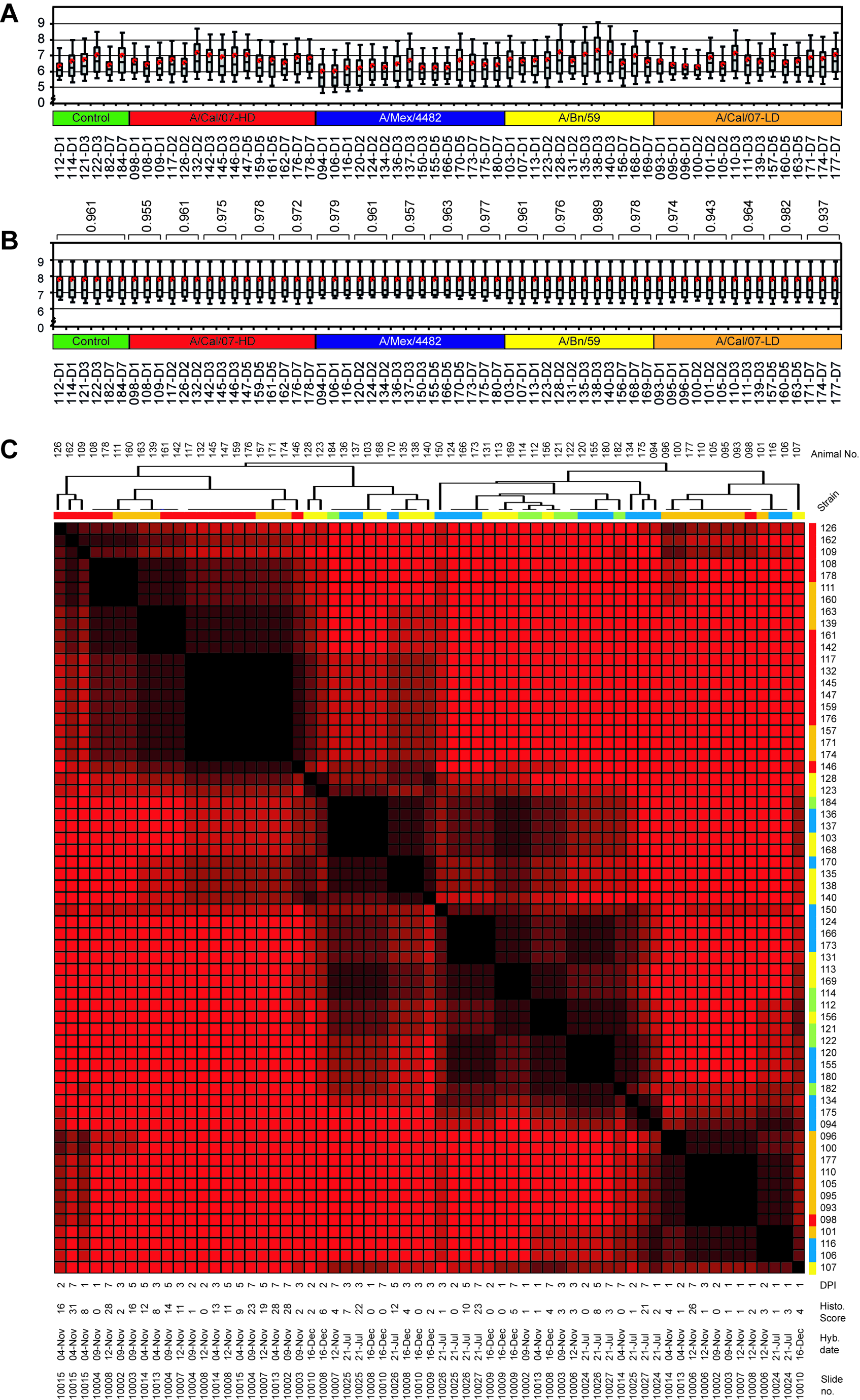

Supplement: Figure S2 — Normalization of microarray data and principal component analysis of samples. Box-and-whisker diagram of the microarray intensities before (A) and after normalization (B). The average correlation coefficient for the samples within each group is shown above the diagram in panel B. Panel C shows the consensus matrix of an unsupervised Nonnegative matrix factorization (NMF) analysis, using the entire data set prior to any statistical comparisons. The color of the heat map indicates the cophenetic correlation used to quantify the robustness of the rank’s evaluation. A strong correlation is indicated as black and weak correlation as red. Strong correlation between the A/Cal/07 infected animals was seen, whereas the other animals did not form any obvious clusters. No obvious clustering could be attributed to be the experimentally introduced variability. (TIF) [file pone.0040743.s002.tif]

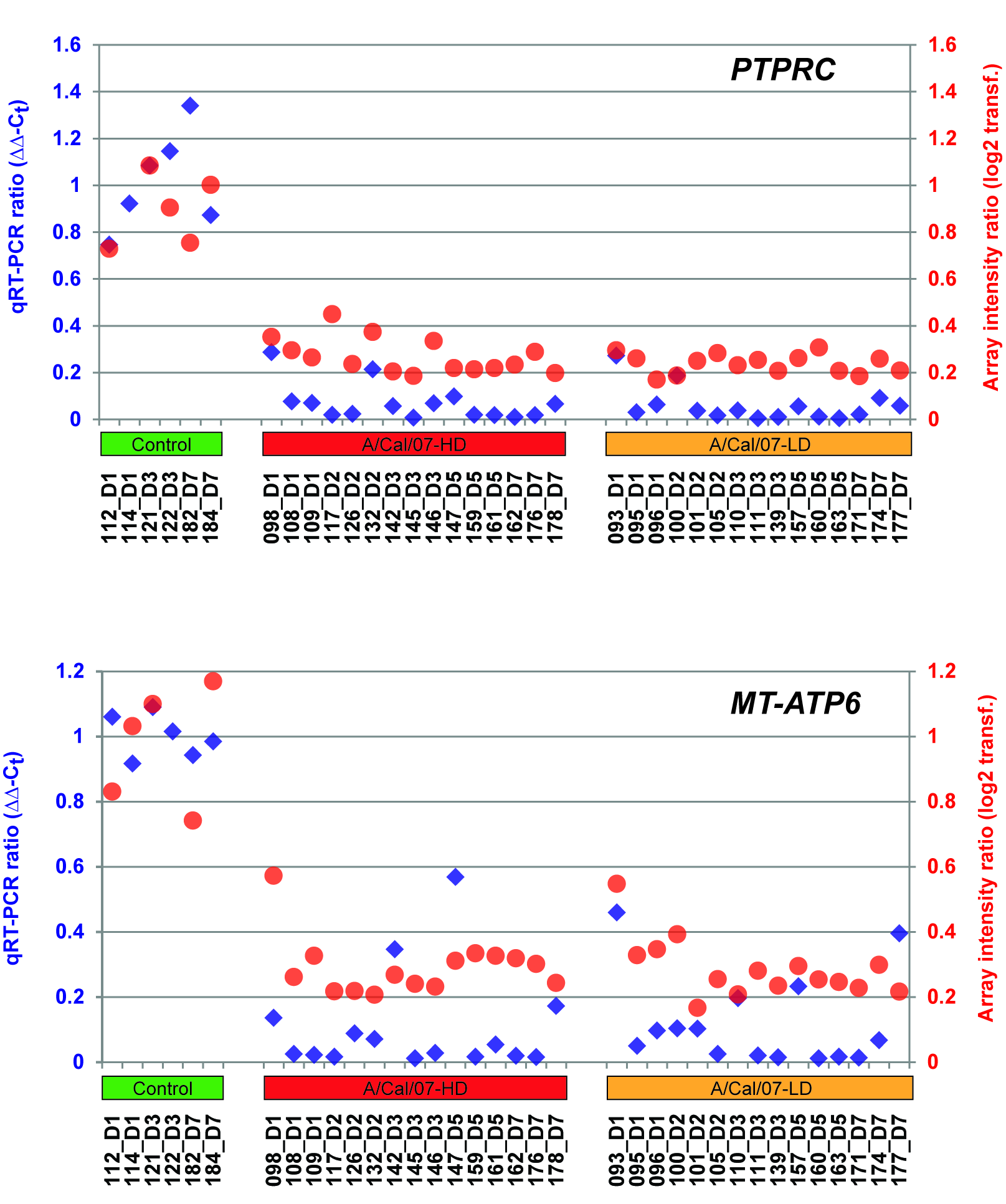

Supplement: Figure S3 — qRT-PCR validation. Scatter plots showing the ΔΔ−Ct values (blue diamonds) and the fold change ratio from the microarray (red circles) for the PTPRC and MT-APT6 genes in control samples and A/Cal/07 infected samples. (TIF) [file pone.0040743.s003.tif]
